# Percutaneous device closure of pediatirc patent ductus arteriosus through femoral artery guidance by transthoracic echocardiography without radiation and contrast agents

**DOI:** 10.1186/s13019-020-01119-w

**Published:** 2020-05-24

**Authors:** Zankai Ye, Zhiqiang Li, Hanlu Yi, Yaobin Zhu, Yan Sun, Pei Li, Ning Ma

**Affiliations:** 1 Department of Cardiac Surgery, Beijing Children’s Hospital, Capital Medical University, National Center for Children’s Health, Beijing, 100045 China; 2 Department of Echocardiography, Beijing Children’s Hospital, Capital Medical University, National Center for Children’s Health, Beijing, 100045 China

**Keywords:** Transthoracic echocardiography, Patent ductus arteriosus, Percutaneous, Device closure

## Abstract

**Background:**

For many years, percutaneous interventional occlusion of congenital patent ductus arteriosus (PDA) has been completed using radiation and contrast agents. In this study, transthoracic echocardiography without radiation and contrast agents was used to complete percutaneous occlusion of pediatric PDA.

**Methods:**

Thirty-two children (8 males and 24 females) with normal heart function and no other intracardiac deformities were diagnosed with PDA (20 funnel type; 12 tube type), One patient had peripheral facial paralysis, 1 patient had epilepsy, and 1 case had multiple cervical deformities. All procedures were performed in the surgical operating room (without Digital Subtraction Angiography (DSA) equipment) under basic anesthesia through the femoral artery pathway. The procedures were guided by transthoracic echocardiography (TTE) by establishing an orbit with a catheter through the femoral artery to thepatent ductus arteriosus,pulmonary artery and right ventricle. A suitable ventricular septal defect occluder was placed using the femoral artery approach,and the treatment effect was evaluated by echocardiography after occlusion. The Outpatient follow-up was performed at 1, 3 months after the operation.

**Results:**

All cases had successful closure of PDA, which took only 35.6 ± 6.4 min. The diameter of the device was 4.8 ± 2.3 mm, and the heart murmur disappeared. There was no shunt between the left pulmonary artery and the descending aortic artery, and the length of hospitalization was 3.4 ± 0.5 days. No other incisions were needed in 32 cases. No occluder was removed, and no residual shunt was found after operation; moreover, no ICU stay was needed, and the mean hospital stay was 3.4 ± 0.5 days. No residual shunt was found at the 1-, 3-month follow-up visit.

**Conclusions:**

PDA closure guided by transthoracic echocardiography via femoral artery puncture is a minimally invasive procedure that avoids injuries due to radiation and contrast agents. This method has wider application prospects in pediatrics.

## Background

Percutaneous interventional catheterization is currently the main method of treating patent ductus arteriosus (PDA) [1, 2], but percutaneous occlusion using radiation has disadvantages. Studies have reported that radiation confers radiation damage to doctors and pediatric patients [3, 4], and the use of contrast agents carries the risk of causing allergies and renal failure [5], especially for children at particular stages of development. There have been reports of PDA occlusion via ultrasound through the femoral artery in China [6]. Transthoracic echocardiography (TTE) completely replaces eliminates the use of radiation during treatment and has become an important method for improving percutaneous interventional technology [7, 8]. This study focused on this specific group of pediatric patients and explored the safety and effectiveness of percutaneous occlusion of PDA in children under transthoracic ultrasound guidance.

## Methods

### Research subjects

From March 2018 to February 2020, 32 children who underwent PDA occlusion through the femoral artery under the guidance of TTE were continuously selected in the Second Department of Cardiology, Beijing Children’s Hospital, National Children’s Medical Center. The selection criteria were as follows: weight ≥ 10 kg, age ≥ 1 year, PDA diameter 2–5 mm, clinical manifestations and cardiac overload:children had varying degrees of palpitations, shortness of breath, and fatigue after exertion, and were susceptible to respiratory infections and growth retardation. During the physical examination, a prominent continuous machine-like noise was heard in the second intercostal space of the left margin of the sternum, accompanied by tremor. The exclusion criteria included children who depended on PDA for survival, right-to-left shunts for severe pulmonary hypertension, infective endocarditis, and other cardiac malformations requiring surgical treatment. The 32 children selected included 8 males and 24 females, with an average age of 5.12 ± 1.32 years and an average weight of 17.32 ± 3.6 kg. Of these cases, 20 PDAs were funnel-shaped with an aortic side diameter of 8.5 ± 2.7 mm and a pumonic diameter was 4.2 ± 0.6 mm. In the 12 tube-type cases, the inner diameter of the PDA was 4.2 ± 1.2 mm, and the length was 5.5 ± 0.8 mm.

### Instruments and materials

A PHLIPS IE33 color Doppler ultrasound system with a probe frequency of 5 ~ 8 MHz (Beijing Huayi Shengjie Technology Co., Ltd. ‘s equilateral umbrella and AGA’s ADOII occluder) was used.

### Surgical methods

The parents of the children signed informed consent before the operations. TTE examinations were performed to determine the shape, inner diameter and length of the PDA.

The patients were supine. After basic anesthesia, the right femoral artery was punctured, a puncture sheath was placed, and then a 5F right heart catheter and an extra stiff guide wire were applied. The descending aortic arch was used to deliver the catheter to pass through the PDA. Guided by TTE, the extrastiff guide wire was manipulated to enter the pulmonary artery through the PDA. Using the short-axis view of the aorta, the guide wire was passed through the pulmonary artery to the right ventricle, and the guidewire confirmed to be located in the main pulmonary artery by TTE. The 5F right heart catheter was retracted and replaced with an occluder delivery sheath. The appropriate device (Beijing Huayi Shengjie Technology Co., Ltd. and AGA ADOII) was then selected., The disc on the pulmonary artery side was released from a safe distance to push the occluder under echocardiographic monitoring, and the sheath was retracted to position the discs close to the PDA. After sensing resistance, the sheath was retracted to release the remaining part of the occluder. TTE was used to visualize the position and shape of the occluder, determine the presence of a residual shunt, and determine the blood flow velocity at the initial point of the left pulmonary artery and the descending aorta. Auscultation showed no murmur. After the occluder push-pull procedure, the occluder was released, and the delivery sheath was withdrawn. Compression hemostasis was performed at the puncture site. All patients were followed up at the outpatient clinic for echocardiography, chest radiographs, and ECG at 1 month, 3 months, after the operation.

### Statistical analysis

Data are expressed as mean ± SD and range. Differeces between groups were compared using unpaired,2-sided stuent t-tests for continuous variables. The chi-square test,or Fisher’s exact test was used to categorize variables. A *p* value of < 0.05 was defined a statistically significant. Data were analyzed using SPSS software (Version22.0).

## Results

All 32 children underwent successful PDA occlusion under TTE guidance. No X-ray examinations or contrast agents were used during the operation. (Table 1) The mean time from completion of puncture to release of the occluder was 35.6 ± 6.4 min, and the diameter of the occluder was 4.8 ± 2.3 mm. Furthermore, there were no residual shunts, and auscultation showed no murmurs. There was no No stenosis was observed in the descending aorta or left pulmonary artery, and no complications occurred, including hemolysis, residual shunt, peripheral vascular injury, or cardiac perforation. The hospital stay was approximately 3.4 ± 0.5 days, and all patients were discharged routinely. No complications occurred in the 32 children who were followed up at 1, 3, and 6 months (Table 2).

**Table 1:** Demographic data of the studied population

	***Cases(n = 32)***	***Controls(n = 28)***	***P value***
***Age(y)***			
***Mean ± SD***	***5.12 ± 1.32***	***5.42 ± 1.24***	***0.831***
***Weight (kg)***			
***Mean ± SD***	***17.32 ± 3.6***	***16.68 ± 2.9***	***0.927***
***Sex***			
***Male***	***8***	***10***	
***Female***	***24***	***18***	
***TTE***	***32***	***28***	
***X-ray***	***0***	***28***	
***Contrast Agents***	***0***	***28***	
***Operation Time***	***35.6 ± 6.4***	***48.6 ± 8.6***	***0.031***
***Device Diameter***	***4.8 ± 2.3***	***4.5 ± 2.6***	***0.943***

**Table 2:** 3-month follow-up clinical,electrocardiographic and echocardiographic evolution in percutaneous closure (before and after PDA closure)

	*Before*	*After*	*P-value*
*Functional Status (NYHA Classification)*			
*NYHA I*	*10*	*24*	*0.01*
*NYHA II*	*20*	*8*	*0.01*
*NYHAIII*	*2*	*0*	*1*
*Echocardiographic Parameters*			
*LV diameter (mm)*	*32.28 ± 9.34*	*25.47 ± 7.34*	*0.02*
*LVEF(%)*	*55.24 ± 5.46*	*59.25 ± 7.18*	*0.02*

*NYHA*:New York Heart Association, *LVEF* left ventricle ecjection fraction, *PDA* patent ductus artersiosus

**Fig. 1:**
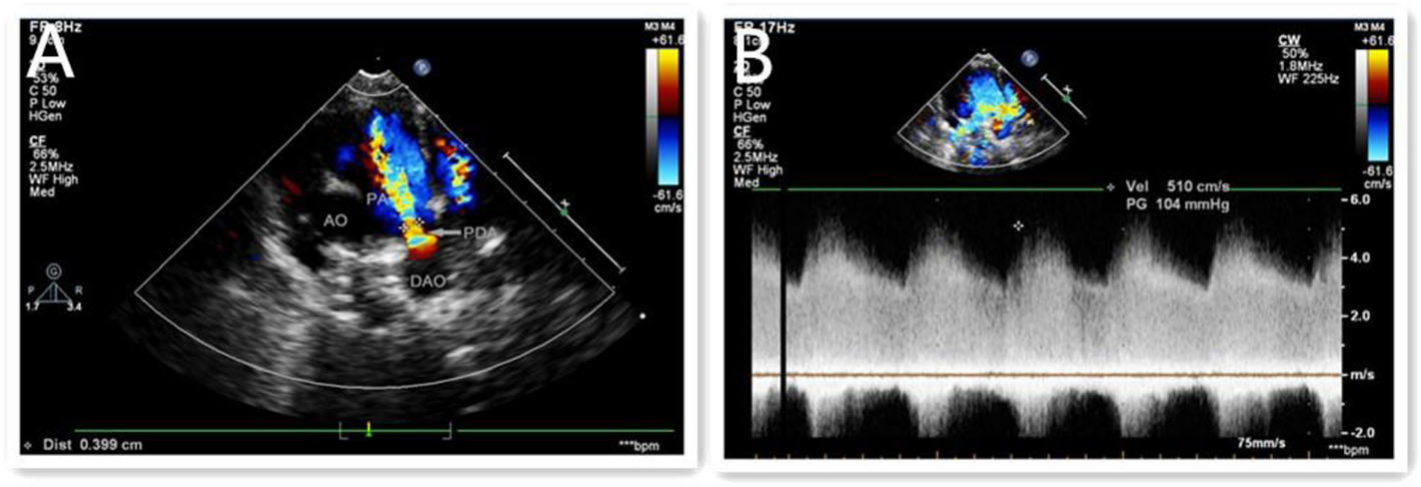
**a**-**b** Pre-procedure: Transthoratic aorta short-axis view shows the PDA (**a**) Continuous Doppler shows an arterial horizontal stunt (**b**)

**Fig. 2:**
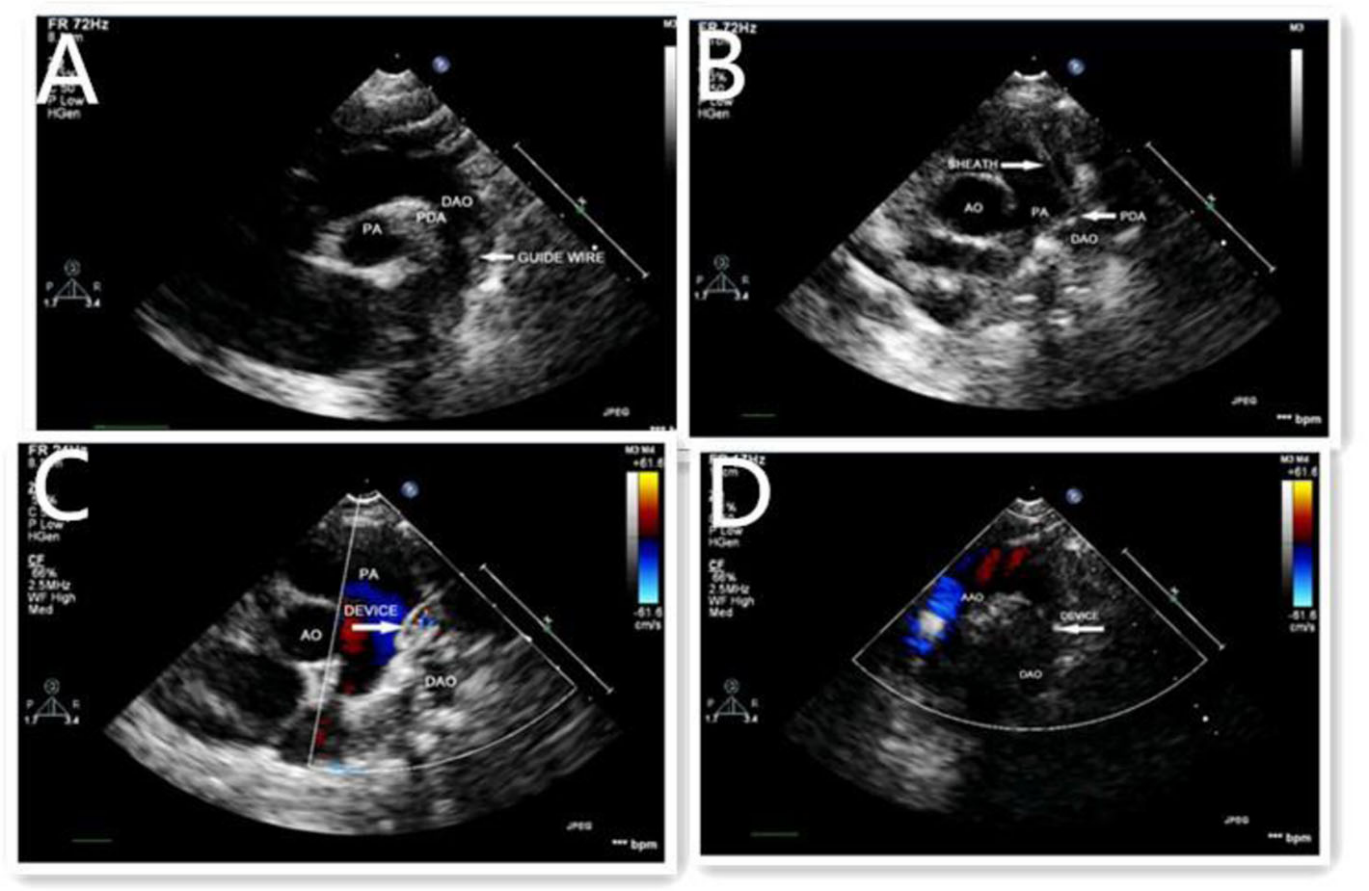
**a**-**d** Intra-procedure: The descending aortic arch nview shows the guide wire passing through the PDA (**a**) The short-axis view of the aorta shows the delivery sheath passing through the PDA into the pulmonary artery, and the dual track sign is visible (**b**) Release of the occluder disc on the pulmonary side (**c**) The descending artic arch view shoes the aortic occluder disc (**d**)

**Fig. 3:**
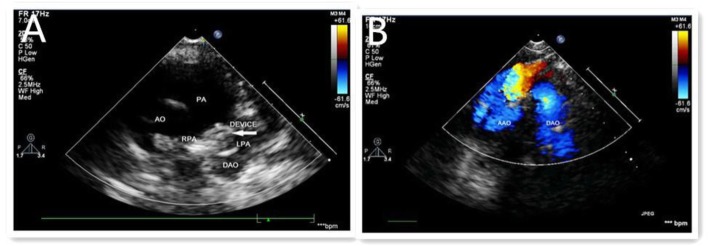
**a**-**b** Post-procedure: The device was fixed without stenosis of the left pulmonary artery (**a**) Descending aorta blodd flow was normal (**b**)

Figures 1, 2 and 3 show the entire process for occlusion of funnel-type PDA under the guidance of transthoracic ultrasound alone.

## Discussion

The current study demonstrated successful transcatheter ductal closure guided only by TTE. Previously published studies have demonstated acceptable reliability of this procedure [9, 10]. Our research focused on PDA closure for pediatric patients under the guidance of TTE alone. As a new technology, this procedure has simplified surgery and also avoids the damage caused by X-rays and contrast agents, especially in children. The operation time was significantly different from that of the control group. However,no X-rays were performed,and no contrast agents were used, We completed the operation more quickly under the premise of fully ensuring the safety of the surgeon and the patient. Time was saved using cardiovascular angiography, making the entire procedure easier and less invasive.

Porstmann et al. [11] first introduced the method of percutaneous treatment of PDA with an occluder in 1967. Because it has the advantage of not requiring a surgical incision, the technology hasrapidly developed and has now become a mainstream treatment method [12–15]. However, during the process of interventional treatment, X-rays and contrast agents must be used, which increases the risk and damage of the treatment [16], especially for children during certain stages of growth and development. In recent years, the use of echocardiography to completely replace X-rays use during interventional therapy has become an important method of improving interventional therapy technology [17]. At present, simple echocardiography-guided atrial septal defect occlusion has become well recognized and is widely carried out [18, 19], resulting in a new method of interventional treatment technology to overcome irradiation injury.

Children with congenital heart disease are a specific patient group. To further overcome the injuries and damage caused by X-rays and contrast agents in children, the Second Department of Cardiology, National Children’s Medical Center, started using only ultrasound guidance without X-ray in March 2018. Percutaneous PDA occlusion requires no thoracotomy, avoids X-ray irradiation, and does not require extracorporeal circulation. All 32 cases were successfully occluded during one operation. The operation time were significantly reduced, and no major complications occurred during follow-up. The overall quality of life of the children was improved by different degrees compared to that before surgery.

The author summarizes the experience as follows:

1. Since none of these children underwent angiography,and the procedure relied entirely on the echocardiography diagnosis;therefore, the ultrasound requirements were higher. We recommend using the S5–1 probe, which has better penetrating power and can clearly show the anatomy of the arterial duct. The disadvantage is that color Doppler will overestimate the blood flow, therefore it is necessary to accurately perform two-dimensional measurements.
2. The surgical indications must be strictly defined to select the appropriate population for surgery. While maintaining safety, we strive to expand the indications to more challenging and difficult cases. The minimum age of this group of children was 1 year and 1 month, and the minimum weight was only 10 kg. Infants, especially those younger than 6 months, have thinner arteries and are more susceptible to vascular damage. The right ventricular outflow tracts in children less than 1 year of age are not long; additionally, the pulmonary artery connection has a certain angle, and the transmission device is easily angled, and these factors increases the difficulty of interventional treatment for PDA. Surgical ligation of very large PDAs in infants and young children may be safer and more effective when a suitable occluder option is not available.
3. The surgeon must have a wealth of cardiac anatomy knowledge experience in interventional therapy and the ability to perform conventional interventional occlusion for PDA under X-ray guidance; team members must be able to perform open-heart cardiac surgery in emergency situations. However,no specific emergency occurred in our group of patients.
4. The appropriate guide wire, catheter and delivery sheath must be selected. We used a 5F right heart catheter and a Cordis MPA1 catheter, and we found that the pruned 5F pigtail catheter was the best option. A COOK 0.035 extrastiff guide wire was used. This guide wire was also used in children with large angles. The COOK-0.035 extra stiff guide wire and delivery sheath were used for the PDA delivery sheath. We found that the AGA delivery sheath has better flexibility and can adapt to large-angle operations. The disadvantage is that it cannot deliver larger occluders.
5. The positioning must be accurate. When the occluder is released, the ultrasound probe is first placed in the third intercostal space of the left margin of the sternum to show the long axis section of the pulmonary artery, which is used to clearly monitor the release of the pulmonary side of the occluder. Then, the ultrasound probe was placed in the upper sternal fossa to show the long axis section of the aortic arch, and the release on the aortic side of the occluder could be clearly monitored. The descending aortic arch of the upper sternal fossa and the short-axis section of the aorta near the sternum are the two most important views in the entire surgical process.

For particularly small (< 2 mm) and special unique PDAs, the author recommends radiational interventional occlusion therapy. Since the sample size of this group was limited and the follow-up time was short, the long-term effects need to be further observed. In addition, the development of this new technology requires the surgeon to be proficient in the hybrid technology of echocardiography diagnosis and congenital heart disease intervention therapy, and higher requirements are necessary for the entire hybrid technology team. After formulating strict surgical indications,and advanced operating specifications and performing strict training of medical staff, the technology has broad development and application prospects.

## Conclusion

We show that percutaneous closure of PDA using only by transthoracic echocardiography TTE without radiation and contrast agents is a safe procedure. This method has wide applications in pediatric patients.

## Data Availability

The datasets used and/or analyzed during the current study are available from the corresponding author on reasonable request.

## Abbreviations

PDA: Patent ductus arteriosus
TTE: Transthoracic echocardiography
AAO: Ascending aorta
PA: Pulmonary artery
DAO: Descending aorta
LPA: Left pulmonary artery
RPA: Right pulmonary artery
